# Association of body mass index and physical activity with the risk of developing type 2 diabetes mellitus in the next decade

**DOI:** 10.1186/1758-5996-7-S1-A155

**Published:** 2015-11-11

**Authors:** Francisca Alexandra Araújo Da Silva, Rosangela Gomes dos Santos, Leandro Araújo Carvalho, Aline Mayra Lopes Silva, Ana Luísa Batista Santos, Thereza Maria Magalhães Moreira

**Affiliations:** 1 Universidade Estadual do Ceará, Fortaleza, Brazil

## Background

The chronic noncommunicable diseases appear as the main causes of death and disability in the world. So, type 2 diabetes is one of the most significant and modifiable degrading situation for health.

## Objective

To investigate the association of body mass index and physical activity with the risk of developing type 2 diabetes.

## Materials and methods

This study was developed during health activities carried out by primary care to 183 city residents members of the interior of Brazilian Northeast. The risk of developing type 2 diabetes in the next 10 yrs. was obtained through the questionnaire Finnish Diabetes Risk Score. It was considered in overweight the users who had a body mass index equal or higher than 25.0, and physically active users who performed a minimum of 150 min of physical activity per week.

## Results

It was found a significant association between body mass index (p <0.00) and physical activity (p <0.01) with the risk of developing type 2 diabetes in the next decade. There was a greater participation of women in the study (121; 66%). In comparison with male gender, female was more physically inactive (105; 57.4%). It was founded a high risk of developing type 2 diabetes in the sample (122, 66.7%).

## Conclusions

The results indicate that there may be interference between distribution of body mass as well as to the practice of physical activity and the risk of developing type 2 diabetes in the next 10 yrs.

